# Supplementary material for: A radical S-adenosyl-L-methionine enzyme and a methyltransferase catalyze cyclopropane formation in natural product biosynthesis
Source: Nat Commun. 2018 Jul 17;9:2771. doi: 10.1038/s41467-018-05217-1 (PMC6050322; doi:10.1038/s41467-018-05217-1)
Supplement: Supplementary file 1 — Supplementary Information [file 41467_2018_5217_MOESM1_ESM.pdf]

Supplementary Information

A radical *S*-adenosyl-L-methionine enzyme and a methyltransferase catalyze cyclopropane formation in natural product biosynthesis

1. Supplementary Figures

Supplementary Figure 1 | The selected enzymatic cyclopropanation strategies in natural product biosynthesis

Supplementary Figure 2 | Construction and verification of the *Δc10Q* mutant strain *S. zelensis* TG1405

Supplementary Figure 3 | Purification and characterization of the radical SAM proteins and methyltransferase

Supplementary Figure 4 | A cryptic biosynthetic gene cluster from *Shewanella woodyi* ATCC 51908 that shows high homology and synergy with that of CC-1065 from *Streptomyces zelensis* NRRL 11183

Supplementary Figure 5 | HR-MS and MS/MS analysis of CC-1065 produced by enzymatic reactions

Supplementary Figure 6 | HR-MS and MS/MS analysis of **7** produced by enzymatic reactions

Supplementary Figure 7 | Comparison of the ^{1}H NMR spectra of substrate **6** and product **7**

Supplementary Figure 8 | Comparison of the H-H COSY spectra of substrate **6** and product **7**

Supplementary Figure 9 | HPLC analysis of the product **7** incubated with C10P and/or C10Q

Supplementary Figure 10 | Isothermal Titration Calorimetry (ITC) analysis of Swoo_2002 and C10Q

Supplementary Figure 11 | Effect of different dithionite concentrations on the production of CC-1065 and **7**

Supplementary Figure 12 | Effect of pH on the production of CC-1065 and **7**

Supplementary Figure 13 | C10P/C10Q-catalyzed production of CC-1065 and **7** with different reduction systems

Supplementary Figure 14 | Effect of the Swoo_2002/C10Q ratio on the production of CC-1065 and **7**

Supplementary Figure 15 | A time-course analysis of C10P/C10Q-catalyzed conversion of **6** into CC-1065 and **7**

Supplementary Figure 16 | Multiple sequence alignment of HemN-like radical SAM enzymes

Supplementary Figure 17 | HPLC analysis of the substrate **6** incubated with C10Q and Swoo_2002 C57A

Supplementary Figure 18 | HPLC analysis of the substrate **6** incubated with C10Q and Swoo_2002 mutant variants

Supplementary Figure 19 | HR-MS analysis of SAH and 5'-dA produced by enzymatic reactions

Supplementary Figure 20 | A time-course analysis of the enzymatic production of 5’-dA, SAH, and **8**
Supplementary Figure 21 | HR-MS analysis of 5’-dA and SAH produced in the enzyme assays using CD_3-SAM instead of SAM
Supplementary Figure 22 | Detection of SAM in boiled C10P, boiled Swoo_2002 and boiled C10Q
Supplementary Fig. 23 | Multiple sequence alignment of selected methyltransferases identified a variant of the SAM-binding motif DxGxNxG for C10Q and a likely His residue for activation of the methyl acceptor group in the substrate **6**
Supplementary Figure 24 | HPLC analysis of the substrate **6** incubated with Swoo_2002 and C10Q mutant
Supplementary Figure 25 | Proposed reaction mechanisms from the intermediate radical **9** to the off-pathway product **7**
Supplementary Figure 26 | Sequence similarity network (SSN) analysis of 150 selected methyltransferases

2. Supplementary Tables

Supplementary Table 1 | Homologues of the pair of C10P and C10Q proteins are encoded by many other biosynthetic gene clusters (BGCs)
Supplementary Table 2 | Strains used in this study
Supplementary Table 3 | Plasmids used in this study
Supplementary Table 4 | Primers (shown from 5’ to 3’) used in this study

3. Supplementary References

1. Supplementary Figures

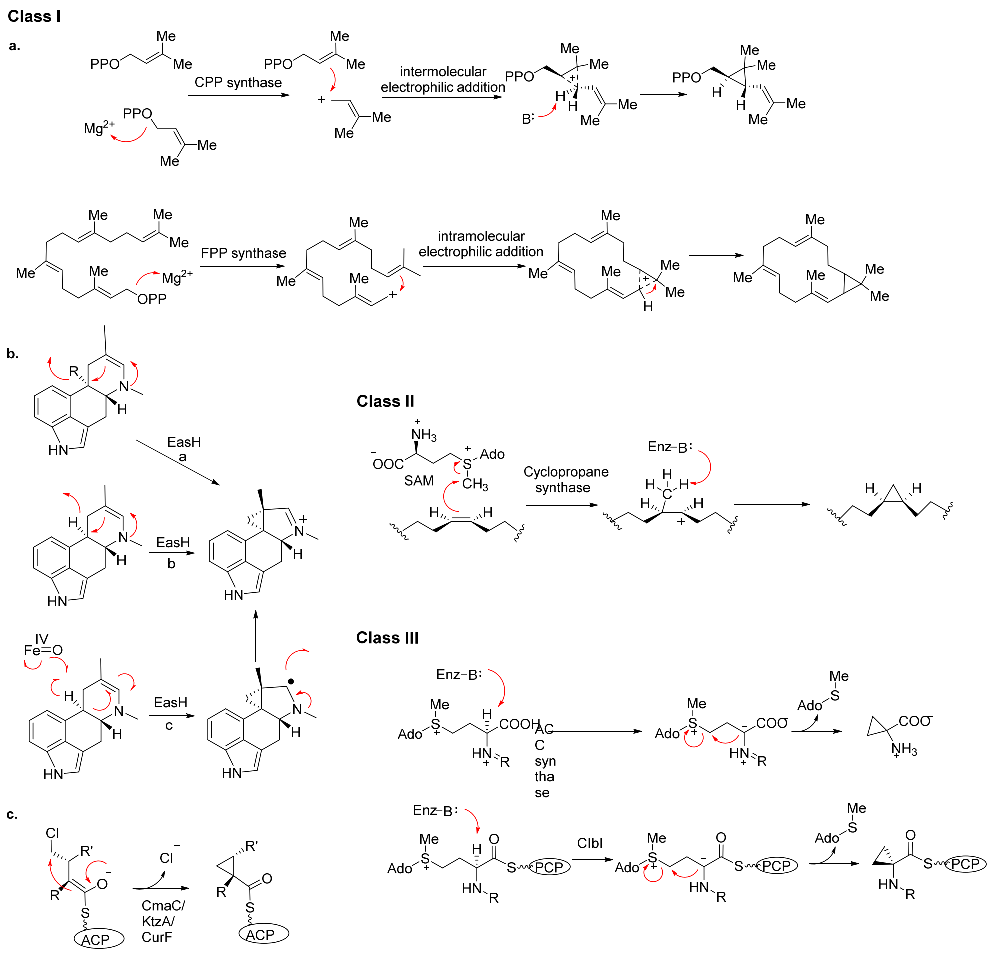

Supplementary Figure 1 | The selected enzymatic cyclopropanation strategies in natural product biosynthesis.

According to the degree of dependence on SAM, these cyclopropanation strategies can be divided into three classes. Class I, **a.** formation of cyclopropane-containing terpenoids via inter- and intramolecular electrophilic addition; **b.** biosynthesis of the alkaloid cycloclavine through an α-ketoglutarate-dependent, non-heme iron oxygenase EasH-catalyzed oxidative rearrangement (three proposed mechanisms); and **c.** construction of cyclopropane-containing building blocks for nonribosomal peptides and hybrid nonribosomal peptide-polyketide compounds using halogenated carrier protein-linked intermediates as the substrates for S_N2-like cyclopropanation. Class II, formation of cyclopropane fatty acids by cyclopropane fatty acid/mycolic acid synthases that catalyze direct transfer of the reactive one-carbon species from SAM to double bonds involving a mechanism of carbocationic intermediates (or transition states). Class III, biosynthesis of 1-aminocyclopropane-1-carboxylate as a precursor to the plant hormone ethylene, and of the cyclopropane warhead of colibactin through a carbanion-induced intramolecular S_N2 reaction mechanism with elimination of methylthioadenosine.

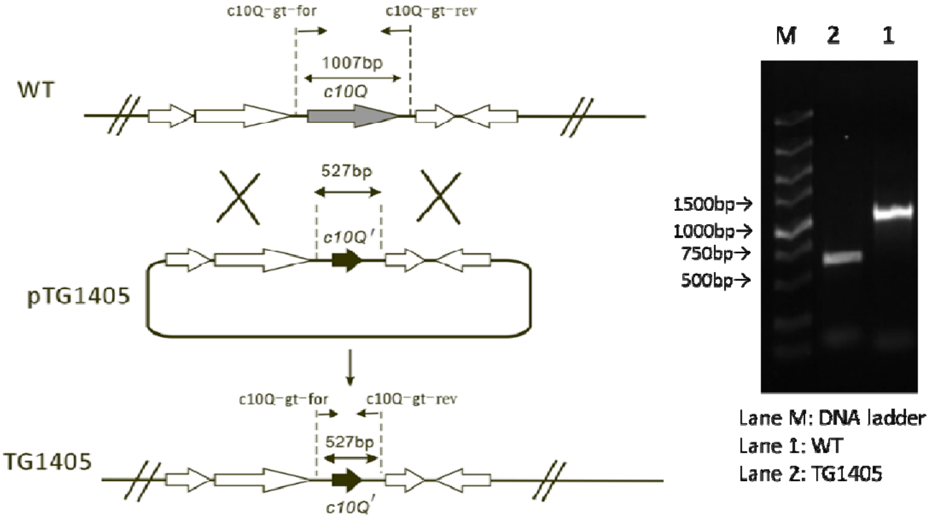

Supplementary Figure 2 | Construction and verification of the *Δc10Q* mutant strain *S. zelensis* TG1405.

Verification was performed by PCR amplification using the genomic DNA from the mutant or wild type strain as the template. The PCR primers are labeled with their predicted sizes of the resulting products.

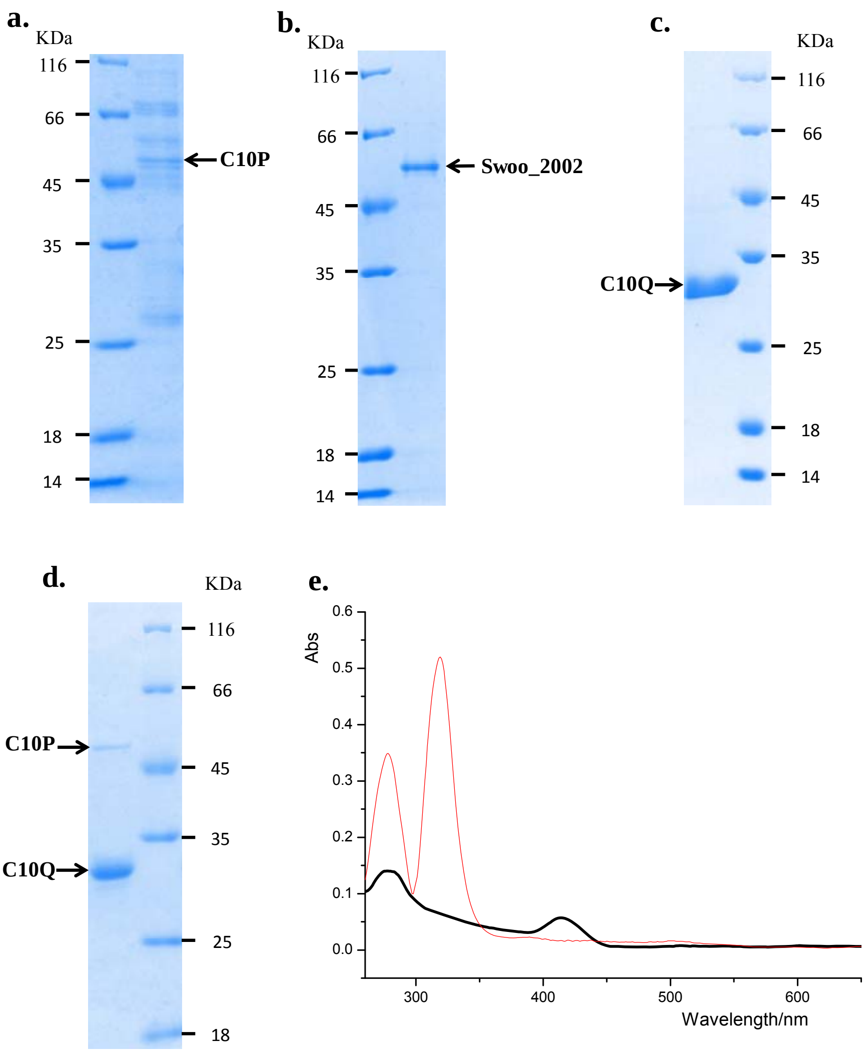

Supplementary Figure 3 | Purification and characterization of the radical SAM proteins and methyltransferase.

a, C10P (51.9 kDa); **b**, Swoo_2002 (51.8 kDa); **c**, C10Q (29.0 kDa); **d**, C10P/C10Q from co-expression; and **e**, ultraviolet-visible (UV-Vis) absorptions of the reconstituted Swoo_2002 (black line) and the protein reduced by sodium dithionite (red line). Proteins C10P and Swoo_2002 were purified under strictly anaerobic conditions and the resulting radical SAM enzymes were reconstituted therein to give dark brownish color. The UV-Vis spectrum exhibited an absorption with A280/A420 ratio of 3.4:1 and an apparent A420 decrease upon reduction by sodium dithionite.

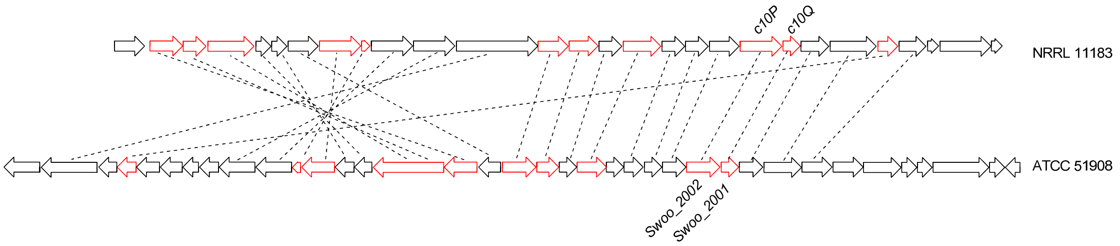

Supplementary Figure 4 | A cryptic biosynthetic gene cluster from *Shewanella woodyi* ATCC 51908 that shows high homology and synergy with that of CC-1065 from *Streptomyces zelensis* NRRL 11183.

C10P shows 67% identity with Swoo_2002.

a.

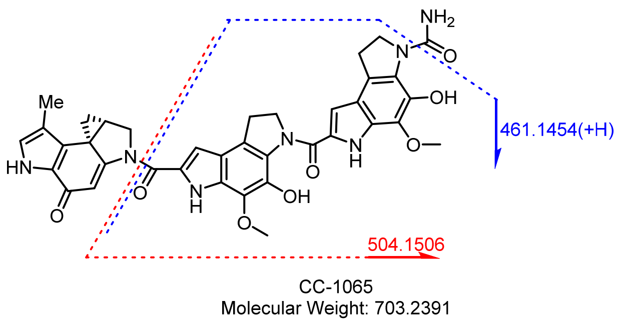

b.

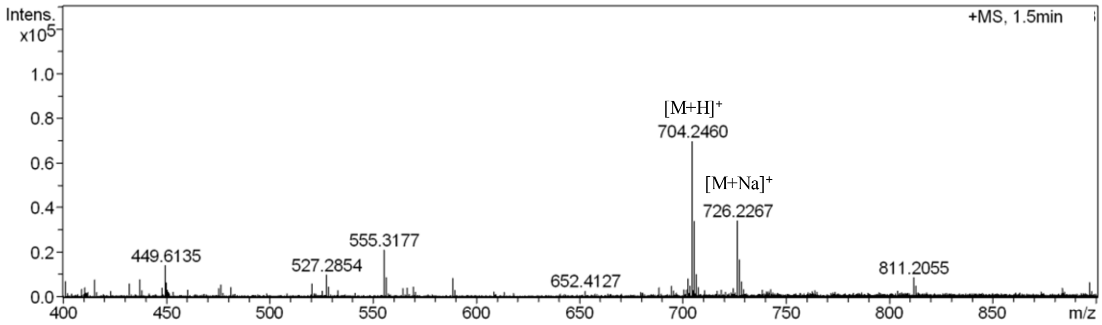

c.

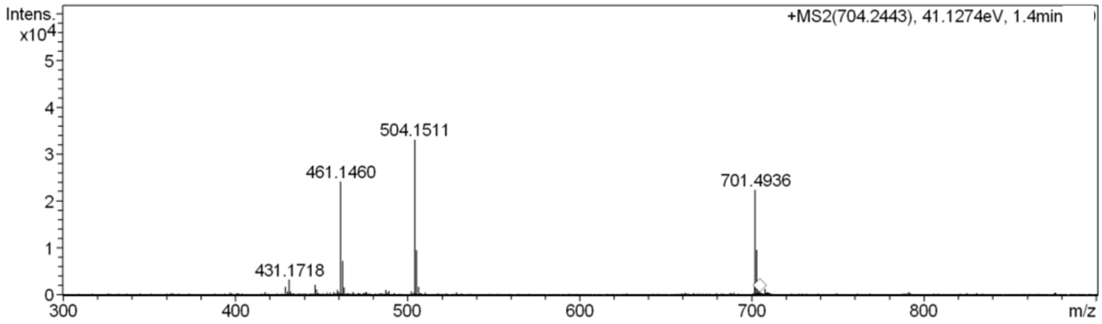

Supplementary Figure 5 | HR-MS and MS/MS analysis of CC-1065 produced by enzymatic reactions.

a, structure of CC-1065. **b**, HR-MS analysis of enzymatically produced CC-1065. **c,** HR-MS/MS analysis of CC-1065.

a.

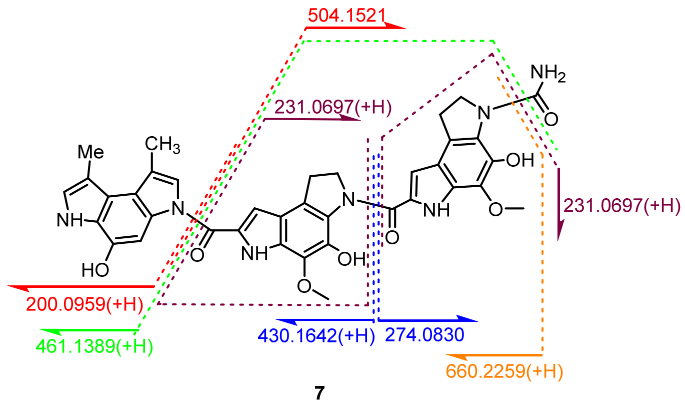

7
Molecular Weight: 703.2391

b.

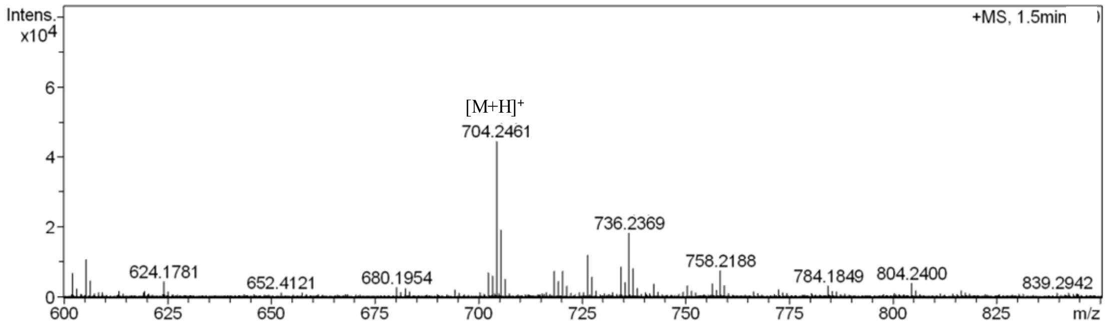

c.

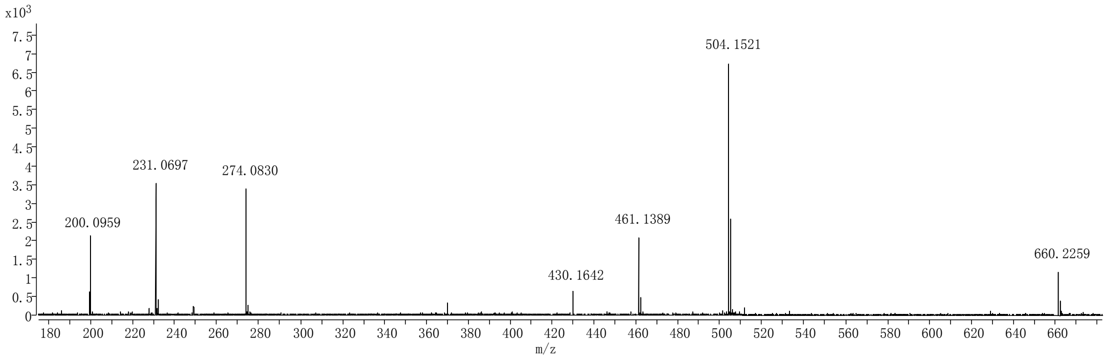

Supplementary Figure 6 | HR-MS and MS/MS analysis of 7 produced by enzymatic reactions.

a, structure of **7**. **b,** HR-MS analysis of enzymatically produced **7**. **c,** HR-MS/MS analysis of **7**.

a.

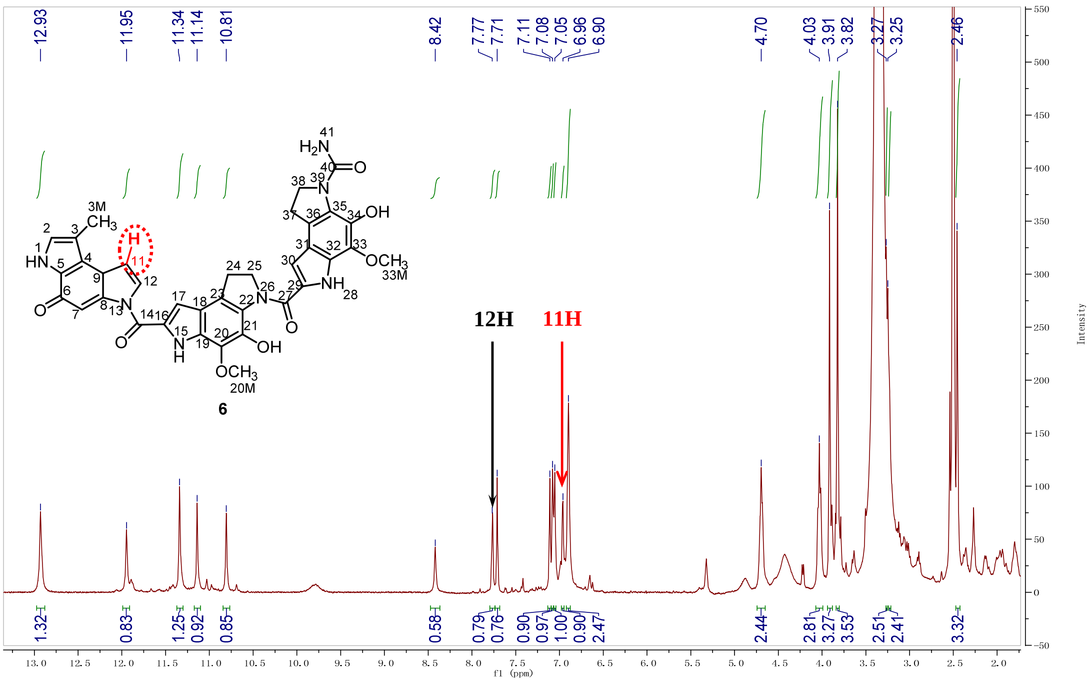

b.

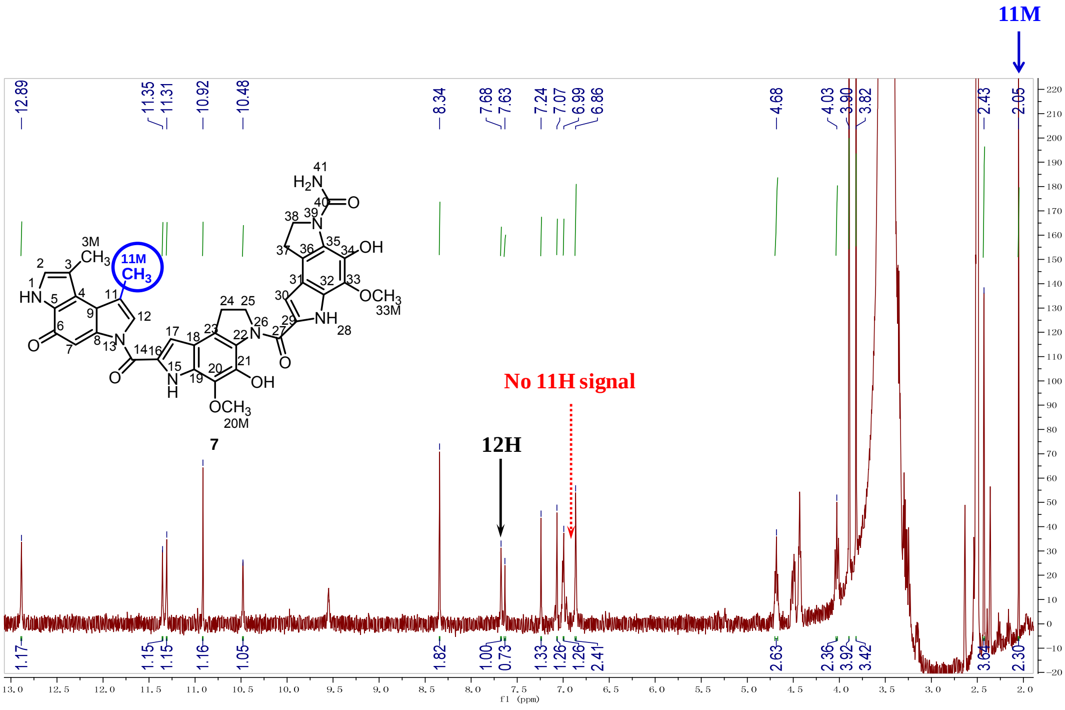

Supplementary Figure 7 | Comparison of the ^{1}H NMR spectra of substrate 6 and product 7. **a,** ^{1}H NMR (600 MHz, DMSO-d_6) spectrum of substrate **6.** The signal 6.96 (d, 1H, J = 2.28 Hz) is the 11-CH of substrate **6** coupling with the adjacent 12-CH. **b,** ^{1}H NMR (600 MHz, DMSO-d_6) spectrum of product **7.** The signal of 11-CH is missing while the signal of 12-CH remains, and the extra signal appears at 2.05 ppm corresponding to the 11-CH_3 connecting with the C=C (11-C).

a.

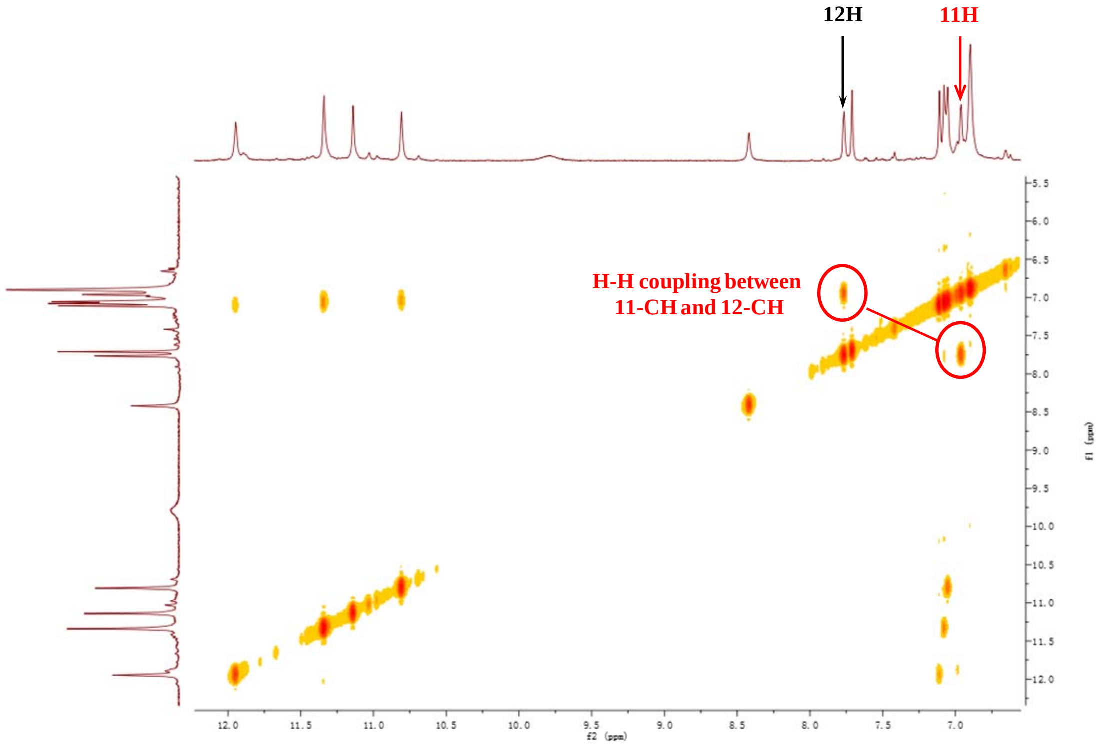

b.

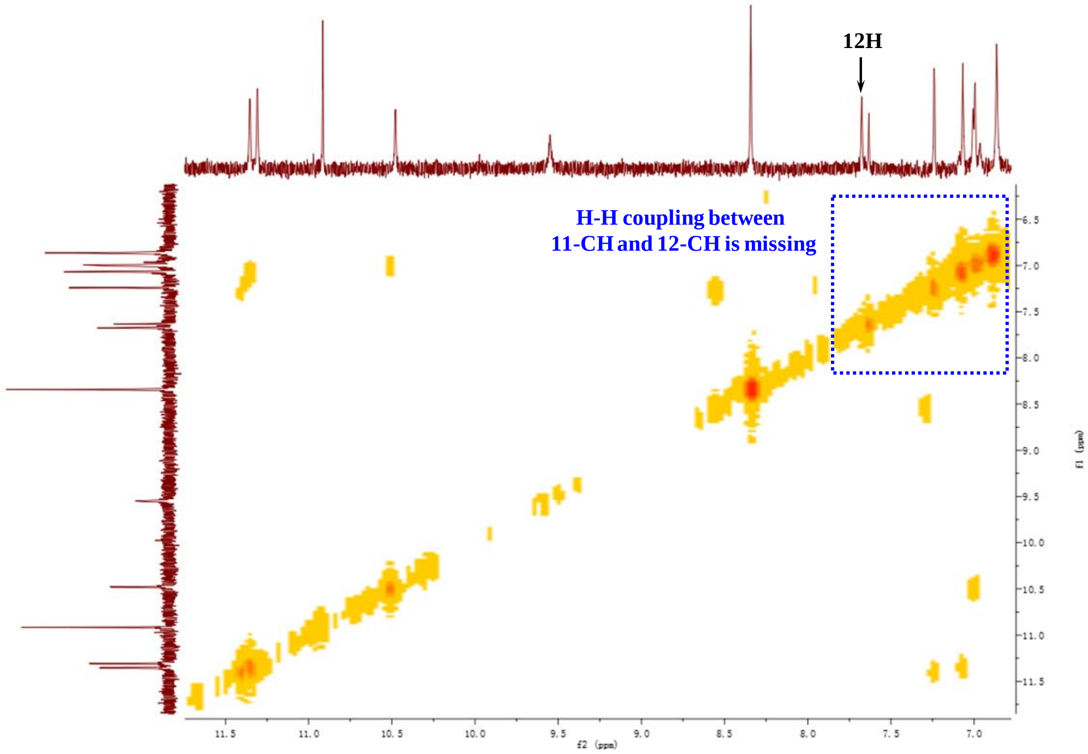

Supplementary Figure 8 | Comparison of the H-H COSY spectra of substrate 6 and product 7. **a,** H-H COSY (600 MHz, DMSO-d_6) spectrum of substrate **6.** There is an obvious signal of H-H coupling between 11-CH and 12-CH. **b,** H-H COSY (600 MHz, DMSO-d_6) spectrum of product **7.** The signal of H-H coupling between 11-CH and 12-CH in substrate **6** is missing.

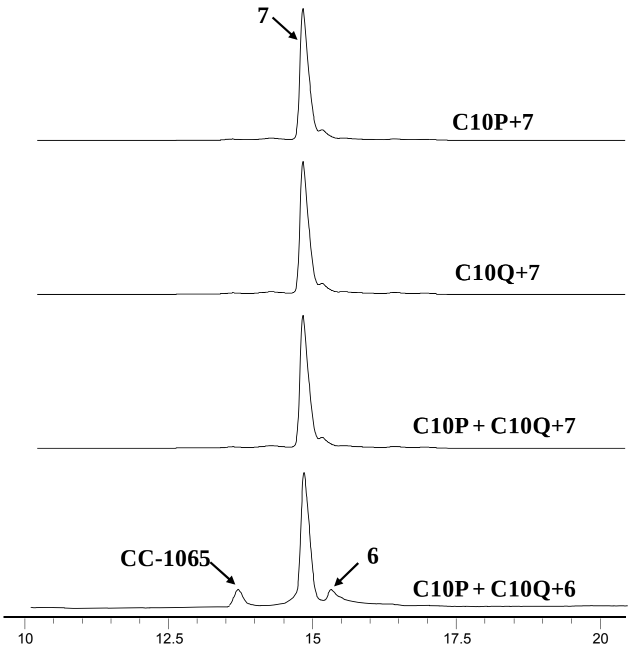

Supplementary Figure 9 | HPLC analysis of the product 7 incubated with C10P and/or C10Q.

The results show that C10P and C10Q can not convert 7 into CC-1065, which indicates that 7 is not a reaction intermediate but an off-pathway product.

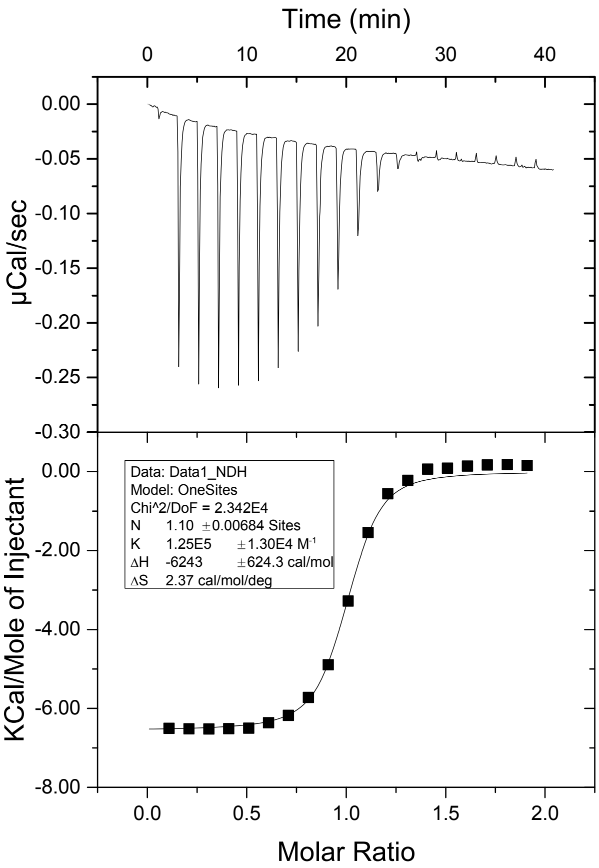

Supplementary Figure 10 | Isothermal Titration Calorimetry (ITC) analysis of Swoo_2002 and C10Q.

The result shows that the Ka value is 1.25×10^5 M^{-1}, indicating that there is a strong protein-protein interaction between Swoo_2002 and C10Q.

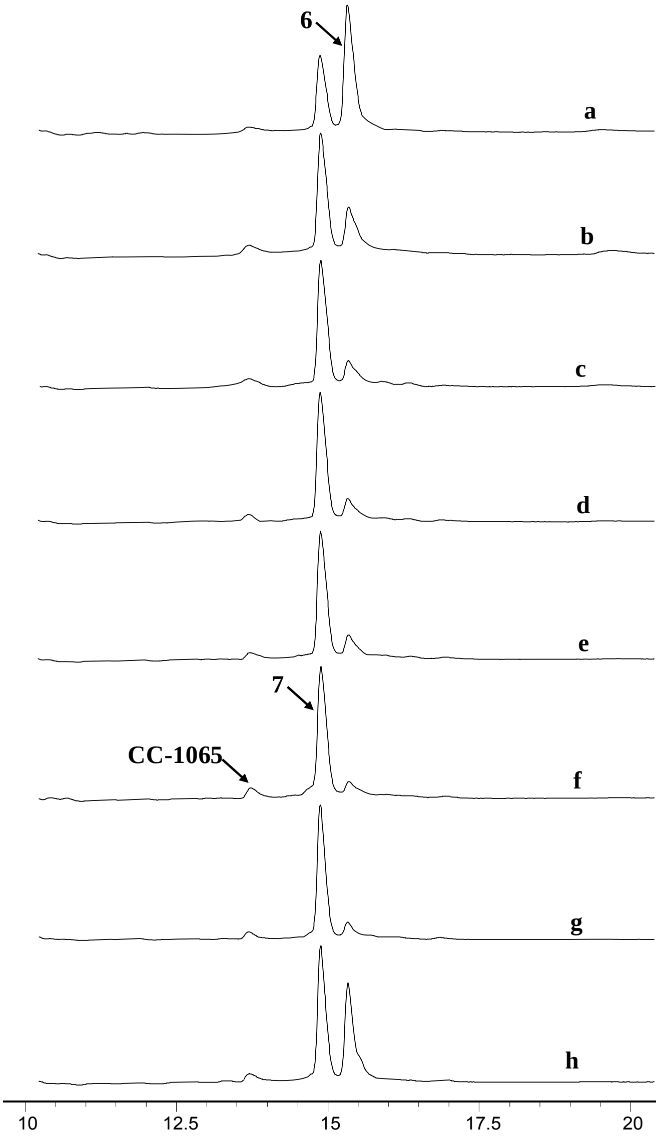

Supplementary Figure 11 | Effect of different dithionite concentrations on the production of CC-1065 and 7.

Samples from **a** to **h** show the concentrations of dithionite at 100 µM (**a**), 200 µM (**b**), 500 µM (**c**), 1 mM (**d**), 2 mM (**e**), 5 mM (**f**), 10 mM (**g**) and 20 mM (**h**), respectively. 5 mM of dithionite is considered as an optimum condition.

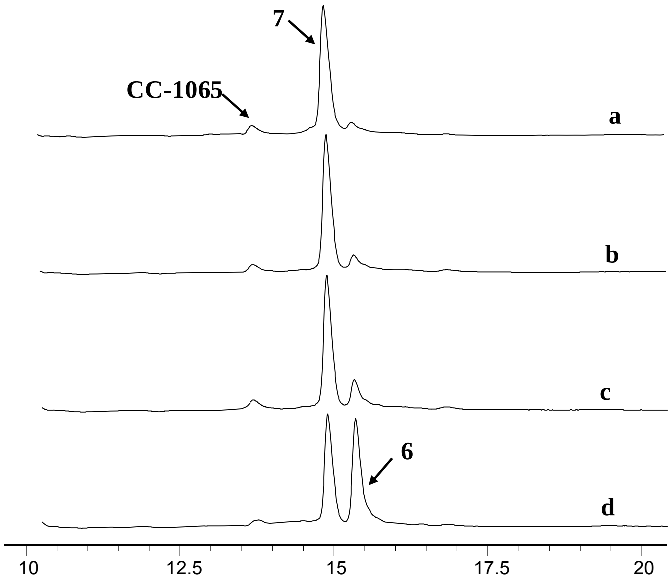

Supplementary Figure 12 | Effect of pH on the production of CC-1065 and 7.

Samples from **a** to **d** show the different pH at 8.0 (**a**), 7.5 (**b**), 7.0 (**c**), and 6.5 (**d**), respectively. pH at 8.0 is considered as an optimum condition.

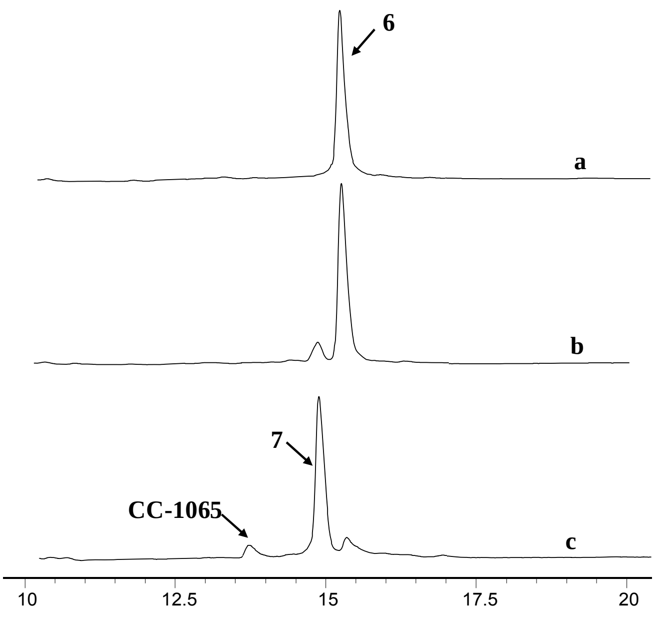

Supplementary Figure 13 | C10P/C10Q-catalyzed production of CC-1065 and 7 with different reduction systems.

(**a**), in the presence of a natural reduction system (flavodoxin, flavodoxin reductase, and NADPH); (**b**), in the presence of the chemical reductant methyl viologen and NADPH; and (**c**), in the presence of the chemical reductant dithionite. Dithionite as the reduction system is considered as an optimum condition.

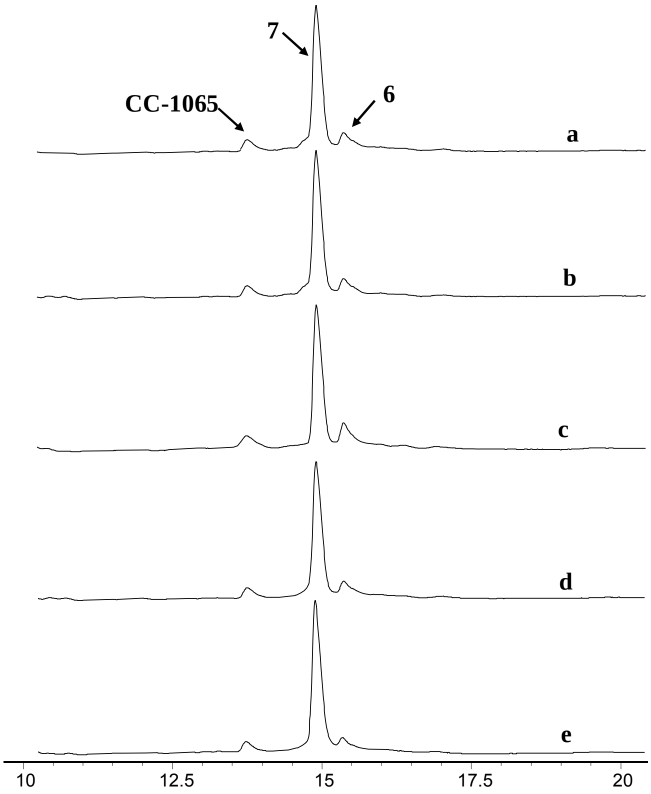

Supplementary Figure 14 | Effect of the Swoo_2002/C10Q ratio on the production of CC-1065 and 7.

Samples from **a** to **e** show the ratio of 3:1 (**a**), 2:1 (**b**), 1:1 (**c**), 1:2 (**d**), and 1:3 (**e**), respectively. The Swoo_2002/C10Q ratio has no detectable influence on the ratio of product CC-1065 and **7**.

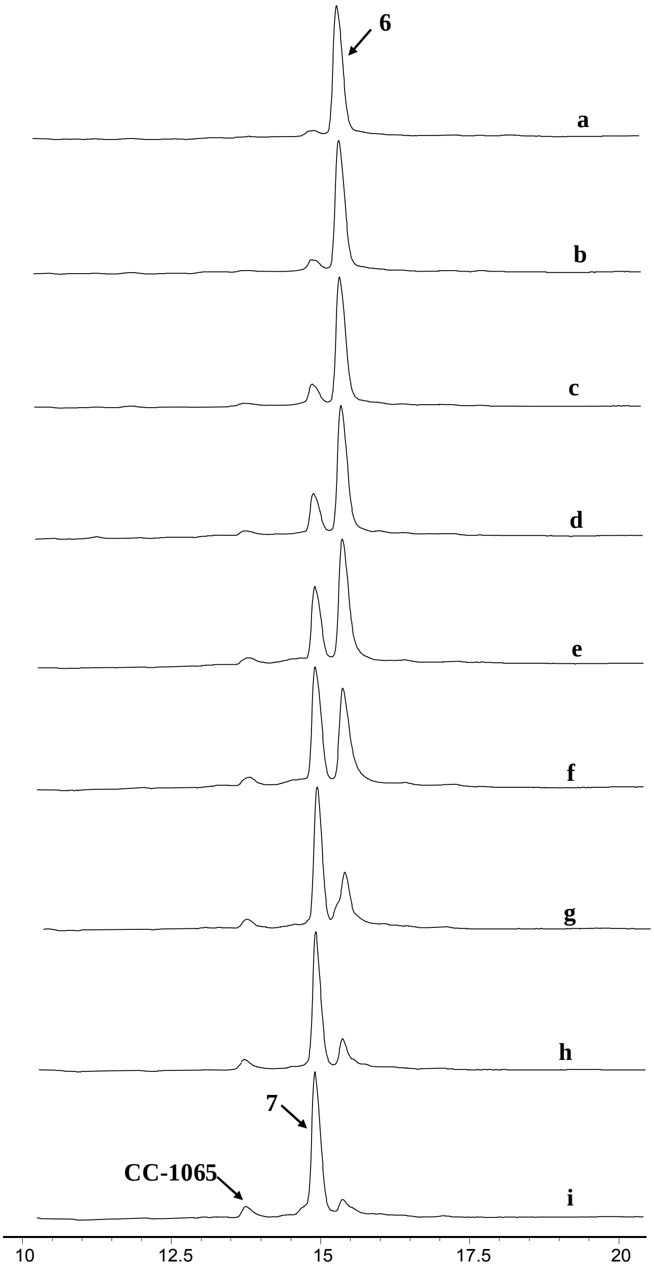

Supplementary Figure 15 | A time-course analysis of C10P/C10Q-catalyzed conversion of 6 into CC-1065 and 7.

Samples from **a** to **i** show the timing at 10 min (**a**), 30 min (**b**); 1 h (**c**), 2 h (**d**), 4 h (**e**), 6 h (**f**), 8 h (**g**), 10 h (**h**), and 12 h (**i**), respectively. Reaction time of 12 h is considered as an optimum condition.

```
HemN            ---MSVQQIDWDLALIQKYNYSGPRYTSYPTA-LEFSEDFGEQ-------AFLQAVARYP  49
C10P            ---------------MSITTTSMPFWKEYPERDNEWVRQYPTKHVAVTEEEVFA------  39
WP_030684647.1  ---------------MSITTTSMPFWKEYPERDNEWVRQYPTKHVPVVEEEVFR------  39
Swoo_2002       -----------------MTLPNLPFWKEYPTRDSEWVRQYPTRHHSIKEEEVFG------  37
YtkT            MTEAPNAAAAAKETITRNFLDTPSFWREYPDRDIEFVRWYPCNIGPLTSDQMFT-TIENR  59
SHJG_8494       ---------MTDTTITRNFLDTPSFWREYPERDIEFVRWYPCDVRPLTADAMYA-QQEKF  50
AQJ11_07385     ---------MTDTTITRNFLDTPSFWREYPERDIEFVRWYPCDVRPLTADAMYA-QQEKF  50
NosN            ------------------------------------------------------------  0

HemN            ERPLSLYVHIPFCHKLCYFCGCNKIVTRQQ-------HKADQYLDALEQEIVHRAPLFAG  102
C10P            PRQMGVYMHIPFCNRLCFSCPYIKFQTERDL--------TLSYLDALKAEITNYAGRPYI  91
WP_030684647.1  RKPMGAYVHIPFCNRLCFSCPYIKHQTDRDL--------TRTYLDALKAEITNYAGRPYV  91
Swoo_2002       KQQMGIYVHIPFCNRLCFSCPYIKHQTDKTI--------TRRYLDALKTEISNYAARPYV  89
YtkT            PKTVSFYLHIPFCNQVCTSCPYNKLHTRNTL--------VTDYLEALKAEILLYSRLGYL  111
SHJG_8494       PKTTSFYVHIPFCNQVCTSCPYNKFNTRNTL--------VRRYIEALKAEIDMYSRLPYL  102
AQJ11_07385     PKTTSFYVHIPFCNQVCTSCPYNKFNTRNTL--------VRRYIEALKAEIDMYSRLPYL  102
NosN            ----MLYVHVPFCHSRCTFCDWVQAIPNKDLLRRPEDSVRRSYIDAMCREIKARGAELSR  56
                      *:*:***:  *  *   :    .             *::*:  **   .

HemN            --RHVSQLHWGGGTPTYLNKAQISRLMKLLRENFQFNADAEISIEVDPREIELDVLDHLR  160
C10P            QDHVITLGYIGGGTPTSLTAQQLDDLLGHLHSSFTFAPDADFSIETTPIDITERKAKVLL  151
WP_030684647.1  QDHEITLGYIGGGTPTALTAPQLDDLLDHLFGSLHMAPDADFSIETTPVDITERKARVLL  151
Swoo_2002       QDHEITLGYIGGGTPTALSSAELEELLGHLHSELNMSDDVELSIETTPIDITKKKAETLY  149
YtkT            DGVKFSSGYFGGGTPTTLRAEQLDDLLGFLRRHLEFTDDYTVTIESTPVDIDQHKIDVLL  171
SHJG_8494       KDVVFTSGYFGGGTPTTLRAEELDDLLGFMKQRLNFSDDCSVTIESTPVDVDQHKLDVLL  162
AQJ11_07385     KDVVFTSGYFGGGTPTTLRAEELDDLLGFMKQRLNFSDDCSVTIESTPVDVDQHKLDVLL  162
NosN            ETHTPYVVMYWGGGTASSLDEREATQIMEALRSSFDFDGIAEATIECSPDTVDAAKLRFFR  116
                        : **** : *   :   :: :   : :      :**  *  :       :

HemN            AEGFNRLSMGVQDFNKEVQRLVNREQ--DEEFIFALLNHAREIGFTSTNIDLIYGLPKQT  218
C10P            DRGVRRISLGVQTFVEEELKNIGRPS--DPEMLKASIRLLQSAGFENINVDLMHGINGQT  209
WP_030684647.1  DRGVRRISLGVQTFVAEELKNLGRPN--DPEMLKNSIRLLRKCGFENINIDLMHGINGQT  209
Swoo_2002       NNGVKRISLGVQTFVQEELESIGRPS--DPKMLENAIKICQEAGFTNINIDLMHGLNGQT  207
YtkT            RHGVNRVSMGVQTFHDPLLRYLGRARAHTGESALRTIELLDRNGMENICIDFMIGIPGQT  231
SHJG_8494       KHGVNRMSLGVQTFHDPLLRYLGRARAHTGESATRTIELIDRSGMENLCIDYMIGIPGQT  222
AQJ11_07385     KHGVNRMSLGVQTFHDPLLRYLGRARAHTGESATRTIELIDRSGMENLCIDYMIGIPGQT  222
NosN            SLGFNRVSSGVQSFDNARLRRLGRRH--SAEQAEHIVYNAREAGFEDVTIDIMSGFPDQD  174
                  *..*:* *** *     . :.*      :     :      *: .  :* : *:  *

HemN            PESFAFTLKRVAELNPDRLSVFNYAHLPT--IFAAQRKIKDADLPSPQQKLDILQETIAF  276
C10P            MEGWEHSLDVAIELGVTCVSFYTYMEFAQ----VSTKRRKLPPVPDRELVDEMFFFAAEK  265
WP_030684647.1  MESWEYSLDVAIELGVTCVSFYTYMEFAQ----VSTKRRKLPPVPDRAVVDDMFLFAADK  265
Swoo_2002       MESWEYSLDKAIELGVTCVSFYTYMEFAQ----VSTKRRKLPPVPTTDVVDEMFMFAAKK  263
YtkT            PELWAQDIATLTSIPVTSFSVYNYAVLPGSEAFFAVQSGITPPCPSTKEADAMYHYMHEE  291
SHJG_8494       HELWAQDIRTLMDLPVTSFSVYNYAVLPGSEVFFALQSGATPPCPSTHEADEMYQYLHTT  282
AQJ11_07385     HELWAQDIRTLMDLPVTSFSVYNYAVLPGSEVFFALQSGATPPCPSTHEADEMYQYLHTT  282
```

```
NosN                AEELDATVAKALELPLSHLSLYSFRPTPG--TFMRRKMAQAE-------KRDYLRKQQLL    225
                     *     :    .:    .*.:.:            :
HemN                LTQSGYQFI--GMDHFARPDDELAVAQRE--------------------GVLHRNFQGYTT   315
C10P                LTQNGF--LGYYGDCFAKPGFQ--------------------------------PKYGETSW   293
WP_030684647.1      LTRHGF--LGYYGDCFAKPGFQ--------------------------------PKYGETSW   293
Swoo_2002           LTENGF--DGYFGDCFAKPGYQ--------------------------------PRYGERSW   291
YtkT                LLSKNY--LALTYNDFAEPMKPEWAAKGAQTFPILPDGSKPFRGLKADSLSLTDHLAQVW   349
SHJG_8494           LMDAGY--LALTYNDFIEPMRPEWEAKGVRTYPVLTDGSKPYRGLETDTFYLTDHLSQVW   340
AQJ11_07385         LMDAGY--LALTYNDFIEPMRPEWEAKGVRTYPVLTDGSKPYRGLETDTFYLTDHLSQVW   340
NosN                FTRARQAIHGSGLTEYASGYFG----------------------------KVSPFAAMYF   257
                    :              :                                      .
HemN                QGDTDLLGMGVSAISMIGDCYAQNQKELKQYYQQVDEQGNALWRGIALTRDDCIRRDVIK   375
C10P                SDDVPIIPLGPTATGHLRDHWYFNEPDLGKYVQIVREGRLPISMGRHIPKDEAIRRSMVL   353
WP_030684647.1      SEDVPIIPLGPTATGHLRDYWYFNEPDIGRYIQTVTAGRLPIAMGKHITKSEAIRRSMVL   353
Swoo_2002           SENIPIIPLGPTATGHLKDHWYFNEPSINKYIEVVNSGQLPISMGQYISKEEAIRRGMIL   351
YtkT                GRCGDMVAIGAGAYGYLNNHLYCTEPDIGRYIETVNSGRLPAVMGAYTDEHERRCRSLVL   409
SHJG_8494           GRCGDMVAMGSGAYGYLNNRMYLTEPDINAYIETCNAGRLPVVMGAYTDEHEQRCRSLVL   400
AQJ11_07385         GRCGDMVAMGSGAYGYLNNRMYLTEPDINAYIETCNAGRLPVVMGAYTDEHEQRCRSLVL   400
NosN                QLRADTVGLGSGAISLLDGRFQSHKKGLLHSYISDP-------LGFDIDVPAGQDRVLVS   310
                          : :*  * . : .     :  :                *          * ::
HemN                SLICNFRLDYSPIEQQWDLLFADYFAEDLKLL--AP----LAKDGLVDVDEKGIQVTA-K   428
C10P                GIKA-GRLNRERFMRLHGVNFLEMFADEIA---------DLVEKGLVTADADGIEVTGPK   403
WP_030684647.1      GVKA-GRVDRETFRRLHGVDFVEMFRAEID---------DLVDKGLITVDDRGIEVTGPK   403
Swoo_2002           GVKA-SKVDRKMFKHLYGVDFVEKFQSEIQ---------DLESKGLVELTQNELKVTDPK   401
YtkT                GLKL-LRVSRADYRARHGVDPYEYFTEKID---------GLVDKGLLEVTDDALQVTYPK   459
SHJG_8494           GLKL-LRMKRSVYRERHGVDPYEYFKDKCD---------DLAAKGLIEITDEAIQVTYPK   450
AQJ11_07385         GLKL-LRMKRSVYRERHGVDPYEYFKDKCD---------DLAAKGLIEITDEAIQVTYPK   450
NosN                LLQAGLAMFDGVLRDQWRLTSGTELAEVLTRPAIAPLSEFLRGRGLV-EDERGIRLPR-K   368
                     :     :          :     :               *   **:      :.:   *
HemN                GRLLIRNICMCFDTYLRQKARMQQFSRVI----------------------------       457
C10P                GWYYLDNISKAFYSPEFRR-YPQHLGADITGFISPQRS--LPLEIVNAGPQGGCHDH       457
WP_030684647.1      GWYYLDNISKAFYSQEYRR-YPQHLGADISHFISSRSL--PLEIINRPSAEEACHDH       457
Swoo_2002           GWYYLDNISKTFYSESFKR-YPQHLGSDITEFQSSVAEWQPIEITTRP---------       448
YtkT                GWHYIDNISKTFYSEANHR-LPQPTSASTEIL-----NWQVRPENGRRLLNIV----       506
SHJG_8494           GWYYIDNICKTFYSDANYR-LPQPTSASTEIL-----NWQIDRADGRKGLPVVQL--       499
AQJ11_07385         GWYYIDNICKTFYSDANYR-LPQPTSASTEIL-----NWQIDRADGRKGLPVVQL--       499
NosN                QAG-ITLIELAFEMAMSQPELE-----------------------------------       389
                        :  *   *
```

Supplementary Figure 16 | Multiple sequence alignment of HemN-like radical SAM enzymes. The selected sequences include HemN from *E. coli* (PDB: 1OLT_A), C10P from the biosynthetic gene cluster of CC-1065, WP_030684647.1 from *Streptomyces* sp. NRRL B-1347, Swoo_2002 from *Shewanella woodyi* ATCC 51908, YtkT from the biosynthetic gene cluster of yatakemycin, SHJG_8494 from *Streptomyces hygroscopicus* subsp. jinggangensis 5008, AQJ11_07385 from

Streptomyces corchorusii strain DSM 40340, and NosN from the biosynthetic gene cluster of nosiheptide. The alignment was carried out using CLUSTAL Omega (1.2.1). The conserved motif CxxxCxxC for binding [4Fe-4S] cluster is marked in green, the conserved glutamine and arginine residues for binding SAM_1 selected for mutation are marked in cyan, and the conserved tyrosine residue for binding SAM_2 selected for mutation is marked in yellow.

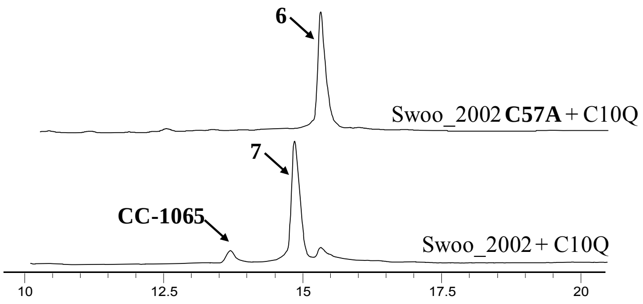

Supplementary Figure 17 | HPLC analysis of the substrate 6 incubated with C10Q and Swoo_2002 C57A.

The Cys-57 is one of the conserved cysteines for binding the iron-sulfur cluster in Swoo_2002. The result shows that the Swoo_2002 C57A mutant lost the ability to catalyze the transformation of **6** into CC-1065 and **7**.

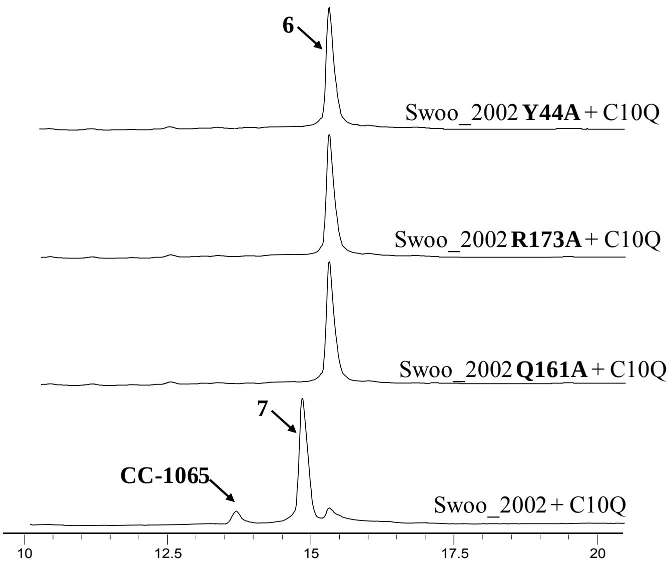

Supplementary Figure 18 | HPLC analysis of the substrate 6 incubated with C10Q and Swoo_2002 mutant variants.

Gln-161 and Arg-173 are two of the conserved residues for binding SAM_1 and Tyr-44 is one of the conserved residues for binding SAM_2 in Swoo_2002. The results show that Swoo_2002 mutants without SAM_1 or SAM_2 binding site lost the ability to catalyze the transformation of **6** into CC-1065 and **7**.

a.

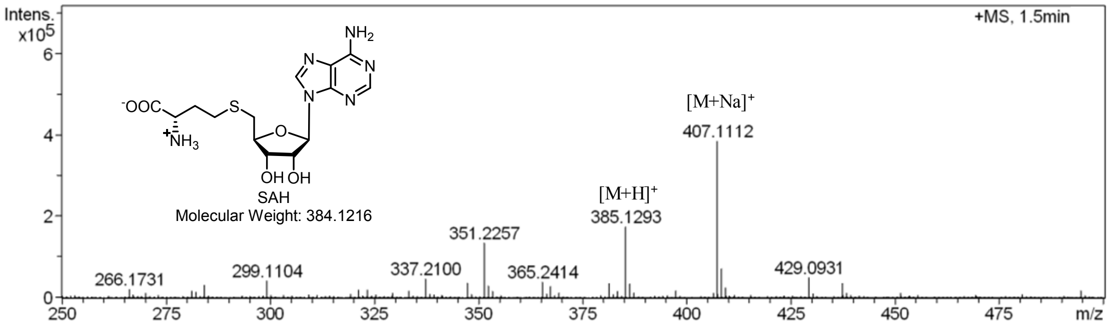

b.

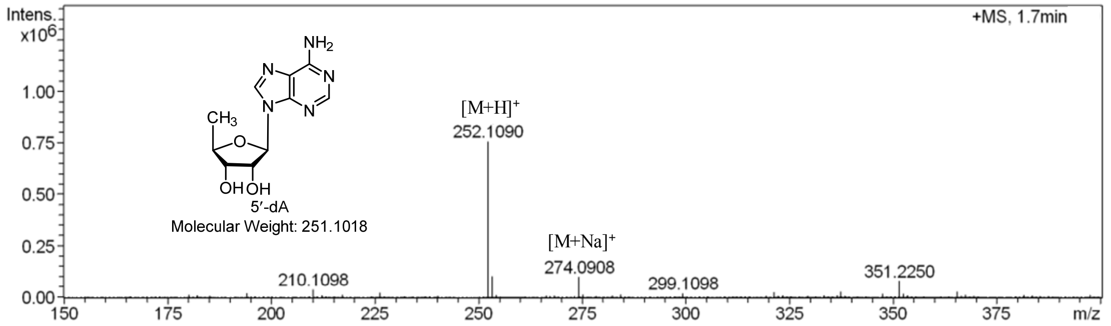

Supplementary Figure 19 | HR-MS analysis of SAH and 5'-dA produced by enzymatic reactions.

a, HR-MS analysis of enzymatically produced SAH. **b,** HR-MS analysis of enzymatically produced 5'-dA.

a.

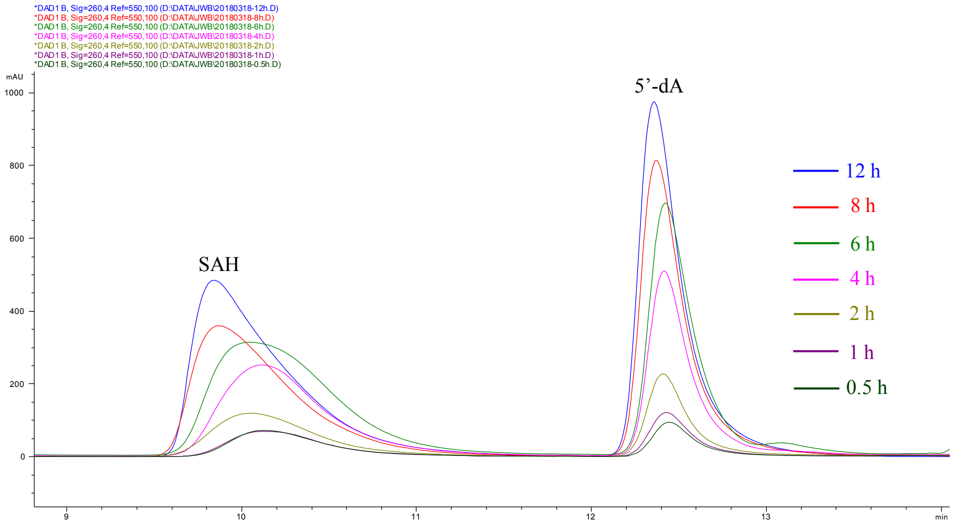

b.

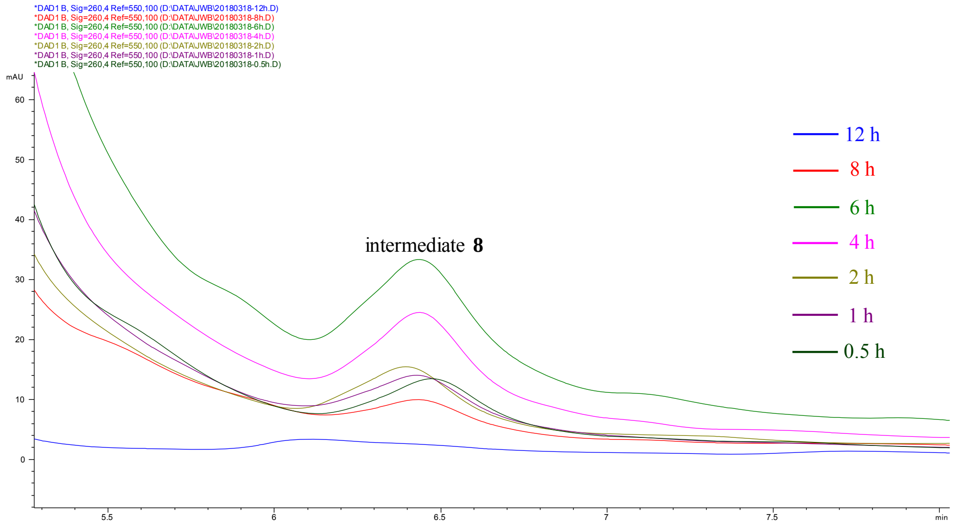

Supplementary Figure 20 | A time-course analysis of the enzymatic production of 5'-dA, SAH, and 8.

a, the profile of the production of 5'-dA and SAH as the reaction proceeded. **b**, the profile of the production of the critical intermediate **8** as the reaction proceeded. The detection wavelength is 260 nm.

a.

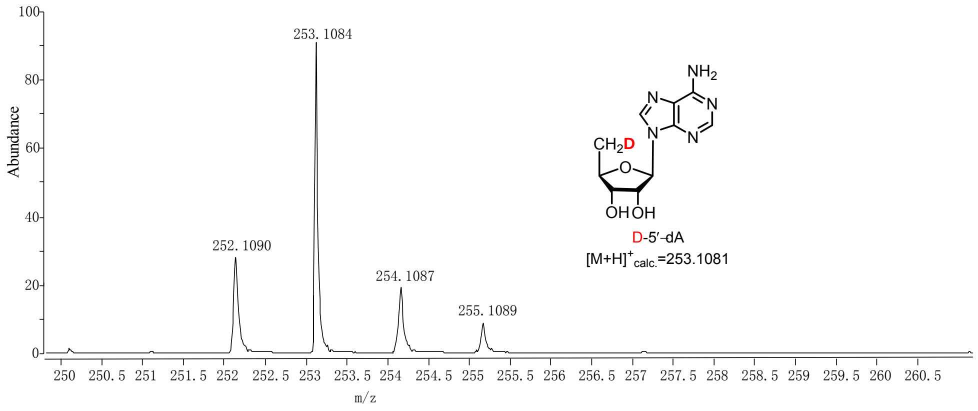

b.

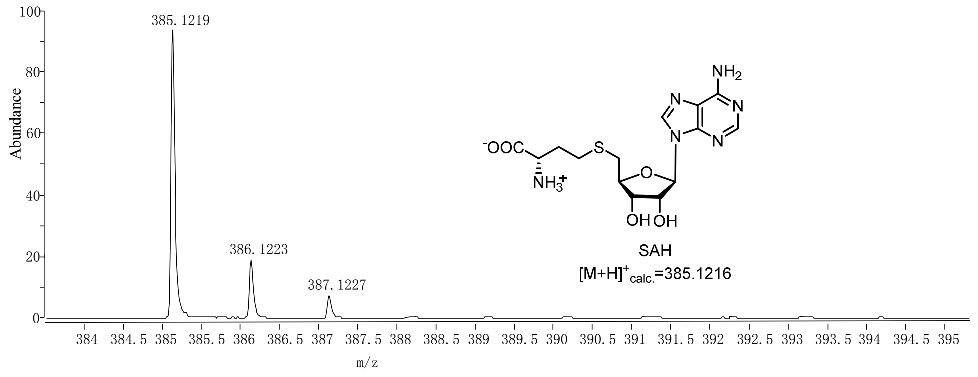

Supplementary Figure 21 | HR-MS analysis of 5'-dA and SAH produced in the enzyme assays using CD_3-SAM instead of SAM.

a, the majority of product 5'-dA shows a mass shift of +1 m/z and the minority remains unchanged.

b, the molecular mass of product SAH remains unchanged.

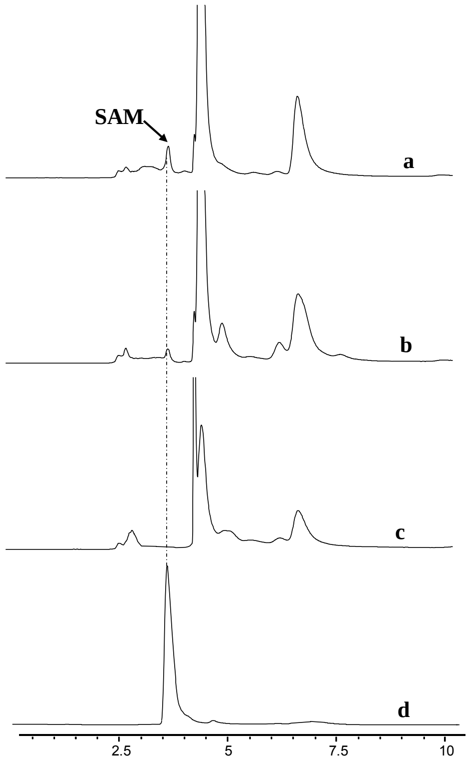

Supplementary Figure 22 | Detection of SAM in boiled C10P, boiled Swoo_2002 and boiled C10Q.

a. boiled C10P, **b**. boiled Swoo_2002, **c**. boiled C10Q and **d**. standard SAM. SAM is detected in boiled C10P and Swoo_2002, but not detected in boiled C10Q.

```
C10Q    ------------------------------------------------------------    0
DnrK    ------------------------MTLTKQDAVNQMMGFFQAKTLTAALDLKLFDHLHNQ    36
MmcR    -----MTVEQTPENPGT-----AARAAAEETVNDILQGAWKARAIHVAVELGVPELLQEG    50
ChOMT   XGNSYITKEDNQISATSEQTEDSACLSAXVLTTNLVY----PAVLNAAIDLNLFEIIAKA    56
IOMT    ------------MASSINGRKPSEIFKAQALLYKHIYAFIDSMSLKWAVEMNIPNIIQNH    48
6OMT    ---------------GAMVMINKENLSSQAKLWNFIYGFADSLVLKSAVQLDLANIIHNH    45

C10Q    ------------------------------------------------------------    0
DnrK    DLSAQ-----AVADKLNSPL------RSVEQMLIALRAMGYLDKQ---------GECYHL    76
MmcR    PRTA--------TALAEATGA---HEQTLRRLLRLLATVGVFDDL-------GHDDLFAQ    92
ChOMT   TPPGAFXSPSEIASKLPASTQHSDLPNRLDRXLRLLASYSVLTSTTRTIEDGGAERVYGL    116
IOMT    GKPI---SLSNLVSILQVPSSKIGN---VRRLMRYLAHNGFFEII-----T-KEEESYAL    96
6OMT    GSPM---TLSELSLHLPSQPVNQDA---LYRVLRYLVHMKLFTKS-----SIDGELRYGL    94

C10Q    -------------------------------------------MTTEAPLLDLAE-RVPL    16
DnrK    PQEHASFLVSS--EPMWLGWLGRHIDTFLYPLWGELNTAVVSDSNQRQKVF--GDNRSWF    132
MmcR    NAL-SAVLLPDPASPVA-TDARFQAAPWHWRAWEQLTHSV---RTGE-ASFDVANGTSFW    146
ChOMT   SXV-GKYLVPDESRGYLASFTTFLCYPALLQVWXNFKEAVVDE--DI-DLFKNVHGVTKY    172
IOMT    TVA-SELLVRGSDLCLA-PMVECVLDPTLSGSYHELKKWIYEE--DL-TLFGVTLGSGFW    151
6OMT    APP-AKFLVKGWDKCML-GAILTITDKDFMAPWHYLKEGILNDGSTS-TAFEKALGTNIW    151
                                                          :

C10Q    DRPHGDIEAVRAYDLGMRRSALMAYKRPTERILAAYREGMTDFVDIGFNTGLLSLHVAGR    76
DnrK    DILYQNPEDVTDFQEFLGKFAAPFI-E--GFIQGYDFSQHHSFLDIGSGIGTLPIAVAES    189
MmcR    QLTHEDPKARELFNRAMGSVSLTEA-G--QVAAAYDFSGAATAVDIGGGRGSLMAAVLDA    203
ChOMT   EFXGKDKKXNQIFNKSXVDVCATEX--KRXLEIYTGFEGISTLVDVGGGSGRNLELIISK    230
IOMT    DFLDKNPEYNTSFNDAMASDSKLIN-L-ALRDCDFVFDGLESIVDVGGGTGTTAKIICET    209
6OMT    DYMAEHPEKNQLFNEGMANDTRLIM-SALVKECSSMFDGITTIVDVGGGTGTAVRNIAKA    210
        :    . :    ::                       .     :*:* . *     :

C10Q    LPDVAVSGVEEFPHLVDVAEDNLTLGVWANMAGDVEFEQSKLSRLPFPDDSADIVYSFSS    136
DnrK    HPDINLTI-CELPQACAFLRDRLAVQGYGDR---INVIEGDVISGKLALQDHDLIHLGWM    245
MmcR    FPGLRGTL-LERPPVAEEARELLTGRGLADR---CEILPGDFFETI--PDGADVYLIKHV    257
ChOMT   YPLIKGIN-FDLPQVIENAPPL-------SG---IEHVGGDXFASV--PQG-DAXILKAV    276
IOMT    FPKLKCIV-FDRPQVVENLSGS-------NN---LTYVGGDMFTSI--PNA-DAVLLKYI    255
6OMT    FPHIKCTV-YDLPHVIADSPGY-------TE---INSIQGDMFKYI--PNA-DAIMMKCI    256
         * :      : *                          ..        :  *

C10Q    LHRWRR--PVETLREAARVCKPD---GTVLIEDVNRLAEE-----GHITFILQ---F-VK    182
DnrK    LHDYAPEIQLTILQNIYQAMPAG---GRFMASETPLNDEKSGPEFT---ALLSLNMLVST    299
MmcR    LHDWDDDDVVRILRRIATAMKPD---SRLLVIDNLIDERPAAS-----TLFVDLLLL-VL    308
ChOMT   CHNWSDEKCIEFLSNCHKALSPN---GKVIIVEFILPEEPNTSEESKLVSTLDNLXF-IT    332
IOMT    LHNWTDKDCLRILKKCKEAVTNDGKRGKVTIIDMVIDKKKDENQVTQIKLLMDVNM--AC    313
6OMT    LHDWDDKECIEILKRCKDAVPRDG--GKVIIIDIILDVKSE-HPYTKMRLTLDLDMM-LN    312
         * :     :  * .   .   .   . .   :     .            :.
```

```
C1OQ       EGGDQFMRSLKAAYSLDQARDLLREAGLDDWHVVEEDLGLV--ISNRPVTPVSM 234
DnrK       DGGIE--------SSTEEYLERFRAVGFENVRIIELS-GPRTLICGDKPQSTNL 344
MmcR       VGGAE--------RSESEFAALLEKSGLRVERSLPCGAGPVRIVEIRRA----- 349
ChOMT      VGGRE--------RTEKQYEKLSKLSGFSKFQVACRAFNSLGVXEFYK------ 372
IOMT       LNGKE--------RNEEEWKKLFIEAGFQHYKISPLT-GFLSLIEIYP------ 352
6OMT       TGGKE--------RTEEEWKKLIHDAGYKGYKITHIS-AVQSVIEAYPY----- 352
            .* :         . .:        *    :
```

Supplementary Fig. 23 | Multiple sequence alignment of selected methyltransferases identified a variant of the SAM-binding motif DxGxNxG for C10Q and a likely His residue for activation of the methyl acceptor group in the substrate 6.

The sequences used are C10Q from the biosynthetic gene cluster of CC-1065, DnrK for carminomycin *O*-methyltransferase from *Streptomyces peucetius*, MmcR for Mitomycin 7-*O*-methyltransferase from *Streptomyces lavendulae*, IOMT for isoflavone-*O*-methytransferase from *Medicago sativa*, ChOMT for Chalcone *O*-Methyltransferase from *Medicago sativa*, and 6OMT for (S)-norcoclaurine 6-*O*-methyltransferase from *Thalictrum flavum* subsp. Glaucum. Sequences are aligned using CLUSTAL Omega (1.2.1), the conserved motif DxGxGxG for binding SAM are marked in green (in C10Q the conserved motif is DxGxNxG instead), and the conserved histidine (His) residue proposed to deprotonate the substrate hydroxyl group is marked in yellow.

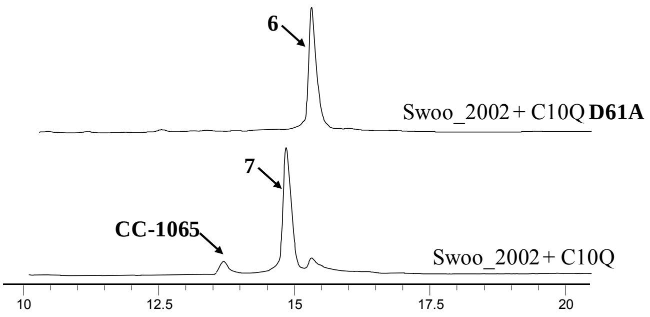

Supplementary Figure 24 | HPLC analysis of the substrate 6 incubated with Swoo_2002 and C10Q mutant.

Asp-61 is one of the conserved amino acid residues for binding SAM in C10Q. The result shows that C10Q mutant without the SAM binding site lost the ability to catalyze the transformation of **6** into CC-1065 and **7**.

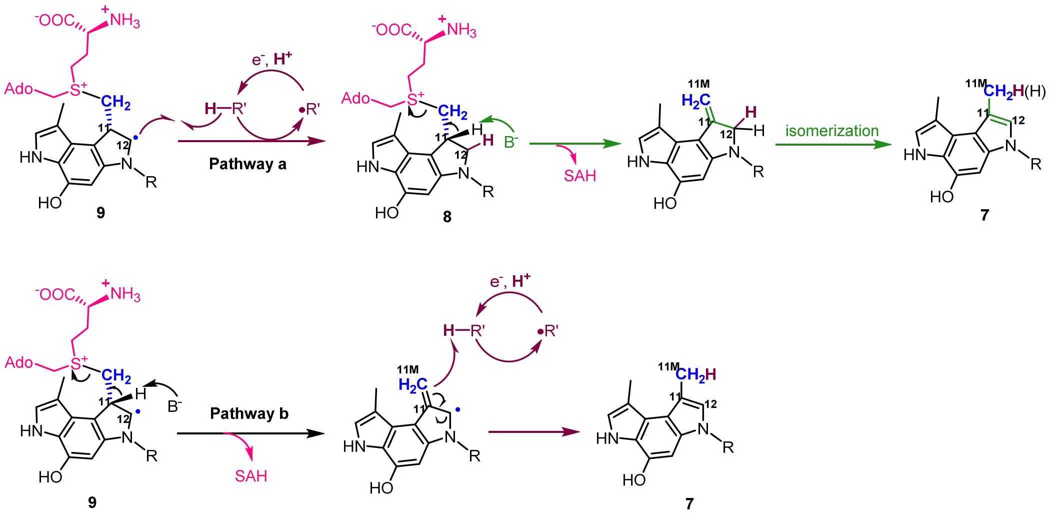

Supplementary Figure 25 | Proposed reaction mechanisms from the intermediate radical 9 to the off-pathway product 7.

In pathway a, the carbon-centered radical at C-12 in **9** abstracts a solvent-exchangeable proton to produce the intermediate **8**. The intermediate **8** may be non-enzymatically converted to the intermediate **10** containing an exocyclic double bond via release of SAH, followed by rapid and thermodynamic driving isomerization to give a methylated off-pathway compound **7**. Pathway b is an alternative proposal for the conversion from **9** to **7**, that is, the carbon-centered radical at C-12 in **9** triggers the removal of a proton at C-11 by an unknown base with elimination of SAH, and then the formed allylic radical is quenched at the C-11M position. However, pathway b is in conflict with our labeling experiments result that the D atom in the produced D-**7** is either located in the C-12 or C-11M position but not merely in C-11M position when using D_2O instead of H_2O in the enzymatic assay.

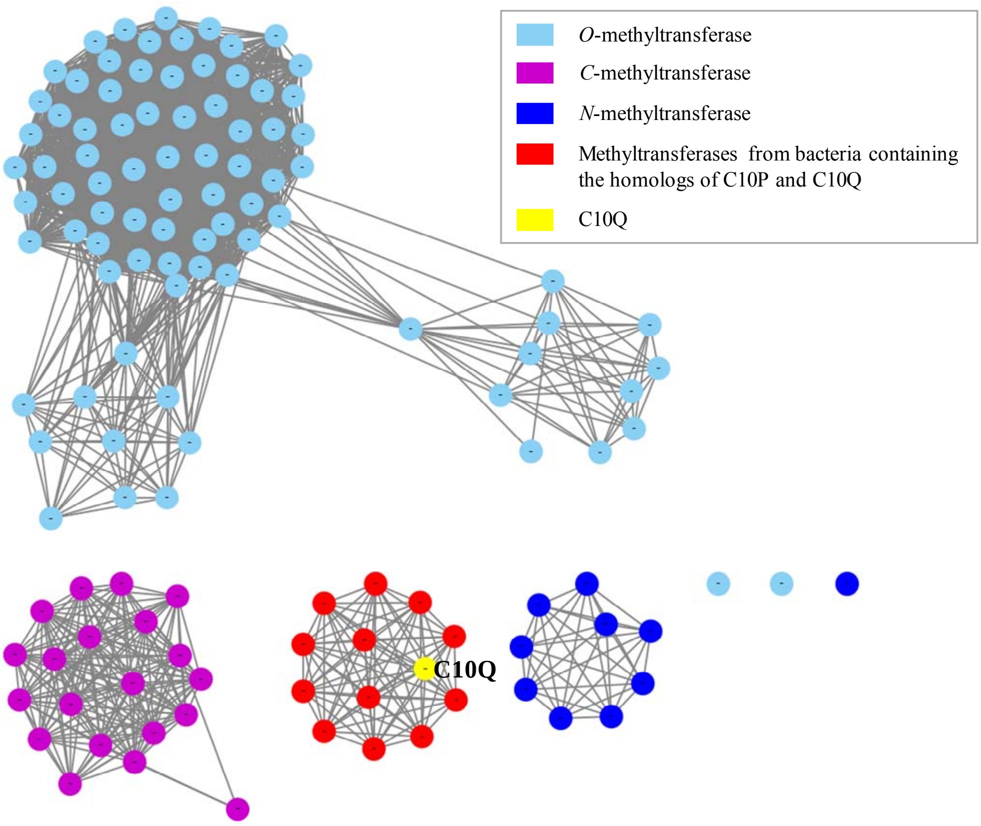

Supplementary Figure 26 | Sequence similarity network (SSN) analysis of 150 selected methyltransferases.

The 150 sequences including *O*-methyltransferases (contain the conserved **D**x**G**x**G**x**G** for SAM-binding and the conserved histidine (His) residue for activating the hydroxy group), *C*-methyltransferases, *N*-methyltransferases, and methyltransferases from bacterial strains containing the homologs of C10P and C10Q. Sequences were used for the construction of sequence similarity network (SSN) from the website (http://efi.igb.illinois.edu/efi-est/stepa.php) [1]. The network parameters used were: (1) E-value, 5; (2) fraction, 1; and (3) alignment score, 30.

2. Supplementary Tables

Supplementary Table 1 | Homologues of the pair of C10P and C10Q proteins are encoded by many other biosynthetic gene clusters (BGCs).

GeneBank accession number	Open reading frames (ORFs)	ORF corresponding to C10P	ORF corresponding to C10Q	Product
JF429418	From ytkA to ytkX	YtkT	YtkU	Yatakemycin
NZ_JOJM01000053.1	From 32187 to 60425 bp	WP_030684647.1	WP_030684646.1	Gilvusmycin
NZ_KQ948212.1	From 11372 to 40755 bp	WP_067124173.1	WP_067124175.1	Gilvusmycin
NC_010506	From Swoo_1990 to Swoo_2028	Swoo_2002	Swoo_2001	Unknown
NC_017765	From SHJG_8481 to SHJG_8515	SHJG_8494	SHJG_8495	Unknown
LMWP01000006	From AQJ11_07275 to AQJ11_07450	AQJ11_07385	AQJ11_07380	Unknown
NZ_KB891296.1	From 958720 to 989508 bp	WP_018510354.1	WP_018510355.1	Potential producer of CC-1065
NZ_CP015098.1	From 269837 to 299239 bp	WP_062924735.1	WP_062924734.1	Potential producer of CC-1065
NZ_KB898279.1	From 26872 to 56268 bp	WP_018891269.1	WP_018891268.1	Potential producer of CC-1065
NZ_JOJB01000017.1	From 77503 to 106904 bp	WP_030847869.1	WP_030847876.1	Potential producer of CC-1065
NZ_KI911520.1	From 163217 to 192616 bp	WP_027735188.1	WP_027735189.1	Potential producer of CC-1065

NZ_JOAW01000206.1 NZ_JOAW01000693.1 NZ_JOAW01000131.1	Discontinuous sequencing	WP_031106456.1	WP_031106457.1	Potential producer of CC-1065
NZ_KB846723.1	From 26856 to 56252 bp	WP_017946733.1	WP_017946732.1	Potential producer of CC-1065
NACY01000136.1	From 1889 to 31303 bp	OSC71384.1	OSC71383.1	Potential producer of CC-1065

The BGC of yatakemycin has been previously cloned in our group[2]. The gilvusmycin has been connected with two potential bacterial strains[2]. Eight of cryptic BGCs have been assigned potential producer of CC-1065[3].

Supplementary Table 2 | Strains used in this study.

**Strains**	**Characteristics**	**Reference**
Streptomyces		
S. zelensis NRRL 11183	Wild type strain, CC-1065 producing	NRRL
S. zelensis TG1402	*C10P* in-frame deletion mutant, CC-1065 non-producing	[4]
S. zelensis TG1405	*C10Q* in-frame deletion mutant, CC-1065 non-producing	This study
S. zelensis TG1406	Mutant TG1405 containing *c10Q* complementary plasmid pTG1406, CC-1065 producing	This study
S. zelensis TG1407	Mutant TG1402 containing *swoo_2002* expression plasmid pTG1407, CC-1065 producing	This study
S. zelensis TG1408	Mutant TG1402 containing *c10P* complementary plasmid pTG1408, CC-1065 producing	This study
S. lividans 1326	Host for gene cluster heterologous expression	
E. coli		
E. coli DH5α	Host for general cloning	Invitrogen
E. coli S17-1	Donor strain for conjugation between *E. coli* and *Streptomyces*	[5]
E. coli BL21(DE3)	Host for protein expression	Invitrogen
E. coli Rosetta(DE3)	Host for protein expression	Invitrogen
E. coli Ro28-P	Rosetta derivative with pTG1409 for expression of C10P	This study
E. coli Ro37-Q	Rosetta derivative with pTG1410 for expression of C10Q	This study
E. coli RoDue-PQ	Rosetta derivative with pTG1411 for co-expression ofC10P/C10Q	This study
E. coli RoTB-SW	Rosetta derivative with pTG1412 for producing Swoo_2002	This study
E. coli RoDue-PQ_M	Rosetta derivative with pTG1413 for producing C10Q-H138A	This study

Supplementary Table 3 | Plasmids used in this study.

Plasmids	Characteristics	Reference
pKC1139	*E. coli-Streptomyces* shuttle vector for gene inactivation with apramycin resistance	[5]
pSET152	gene complementary vector	[5]
pTG1402	pKC1139 derivative for gene replacement of *c10P*	[4]
pTG1405	pKC1139 derivative for gene replacement of *c10Q*	This study
pTG1406	pSET152 derivative for gene complementary of *c10Q*	This study
pTGL407	pSET152 derivative for gene heterologous complementary of *c10P* using *swoo_2002*	This study
pTG1408	pSET152 derivative for gene complementary of *c10P*	This study
pET28a	vector for expression of protein in *E. coli*	Novagen
pET37b	vector for expression of protein in *E. coli*	Novagen
pRSFDuet	vector for co-expression of protein in *E. coli*	Novagen
pRSETB	vector for expression of protein in *E. coli*	Novagen
pTG1409	pET28a derivative for encoding C10P	This study
pTG1410	pET37b derivative for encoding C10Q	This study
pTG1411	pRSFDuet derivative for encoding C10P and C10Q	This study
pTG1412	pRSETB derivative for encoding Swoo_2002	This study
pTG1413	pRSFDuet derivative for encoding C10Q H138A	This study

Supplementary Table 4 | Primers (shown from 5’ to 3’) used in this study.

Primers	Sequence	Usage
c10Q-L-for	ATAAAGCTTGGCTCGTCCTCGAAACCCA	*c10Q* in-frame deletion
c10Q-L-rev	ATACTCGAGCATGCCCAGGTCGTAGGCC	*c10Q* in-frame deletion
c10Q-R-for	ATACTCGAGGCCGCGTACTCGCTCGACC	*c10Q* in-frame deletion
c10Q-R-rev	ATAGAATTCCCACGAACTCCGGAGTCAC	*c10Q* in-frame deletion
c10Q-gt-for	CACGGCGTCAACTTCCTGG	Δ*c10Q*genotype verification
c10Q-gt-rev	TTGGACTCCATCTGGACCACC	Δ*c10Q*genotype verification
C10P-com-for	GTTTTCTAGAGGAGGAGCCAGCATGAGCATCA CCACCACCAG	*c10P* complementation
C10P-com-rev	GTTTGAATTCTTAGTGGTCATGGCAGCCTCC	*c10P* complementation
C10Q-com-for	CATATGAGTTCCGCCGCTATCCGC	*c10Q* complementation
C10Q-com-rev	GAATTCTCACATGCTCACCGGGGT	*c10Q* complementation
swoo_2002-com-for	CATATGATCTTGTCATCTGTCGTTA	*swoo_2002* complementation
swoo_2002-com-rev	GAATTCATCAGCACTGATGTCTTAT	*swoo_2002* complementation
c10P-for	ATAGAATTCCATATGAGCATCACCACCACCAG	expression of C10P
c10P-rev	ATAAAGCTTACTCGAGGTGGTCATGGCAGCCTC C	expression of C10P
c10Q- for	ATAGAATTCGATGACCACTGAGGCACCA	expression of C10Q
c10Q-rev	ATA AAGCTTTCACATGCTCACCGGGG	expression of C10Q
swoo_2002-for	ATAGGATCCACATATGACATTACCTAATTTACCA TT	expression of Swoo_2002
swoo_2002-rev	ATAGAATTCTCAAAGCTTTGGTCGAGTTGTGAT TTCAA	expression of Swoo_2002
c10P –gong-For	ATAGAATTCCATATGAGCATCACCACCACCAG	co-expression of C10P/C10Q
c10P –gong-Rev	ATAAAGCTTACTCGAGGTGGTCATGGCAGCCTC C	co-expression of C10P/C10Q
c10Q –gong-For	ATAGAATTCGATGACCACTGAGGCACCACT	co-expression of C10P/C10Q
c10Q –gong-Rev	ATAAAGCTTTCACATGCTCACCGGGG	co-expression of C10P/C10Q
c10P-Q H138A for	ATCGTCTACTCGTTCTCGTCGCTG**GCC**CGCTGG CGCCGGCCGGTGGAGA	expression of C10Q H138A
c10P-Q H138A rev	TCTCCACCGGCCGGCGCCAGCG**GGC**CAGCGAC GAGAACGAGTAGACGAT	expression of C10Q H138A

Restriction sites are underlined.

3. Supplementary References

1 Gerlt, J. A. *et al.* Enzyme Function Initiative-Enzyme Similarity Tool (EFI-EST): a web tool for generating protein sequence similarity networks. *Biochim. Biophys. Acta* **1854**, 1019-1037 (2015).

2 Huang, W. *et al.* Characterization of yatakemycin gene cluster revealing a radical S-adenosylmethionine dependent methyltransferase and highlighting spirocyclopropane biosynthesis. *J. Am. Chem. Soc.* **134**, 8831-8840 (2012).

3 Wang, X. *et al.* Bioinformatics-guided connection of a biosynthetic gene cluster to the antitumor antibiotic gilvusmycin. *Acta Biochim. Biophys. Sin.* **50**, 516-518 (2018).

4 Wu, S. *et al.* Unified biosynthetic origin of the benzodipyrrole subunits in CC-1065. *ACS Chem. Biol.* **12**, 1603-1610 (2017).

5 Kieser, T. *et al*. *Practical Streptomyces Genetics* (John Innes Foundation, Norwich, UK, 2000).
